# Priorities for Research on Equity and Health: Towards an Equity-Focused Health Research Agenda

**DOI:** 10.1371/journal.pmed.1001115

**Published:** 2011-11-01

**Authors:** Piroska Östlin, Ted Schrecker, Ritu Sadana, Josiane Bonnefoy, Lucy Gilson, Clyde Hertzman, Michael P. Kelly, Tord Kjellstrom, Ronald Labonté, Olle Lundberg, Carles Muntaner, Jennie Popay, Gita Sen, Ziba Vaghri

**Affiliations:** 1 World Health Organization Regional Office for Europe, Copenhagen, Denmark; 2 Department of Epidemiology and Community Medicine and Institute of Population Health, University of Ottawa, Ottawa, Canada; 3 World Health Organization, Geneva, Switzerland; 4 School of Public Health, University of Chile, Santiago, Chile; 5 University of Cape Town, Cape Town, South Africa, and London School of Hygiene and Tropical Medicine, London, United Kingdom; 6 Human Early Learning Partnership (HELP), University of British Columbia, Vancouver, Canada; 7 Centre for Public Health Excellence, National Institute for Health and Clinical Excellence, London, United Kingdom; 8 National Centre for Epidemiology and Population Health, Australian National University, Canberra, Australia; 9 Department of Epidemiology and Community Medicine and Institute of Population Health, University of Ottawa, Ottawa, Canada; 10 Centre for Health Equity Studies, Stockholm, and Department of Health Sciences, Mid Sweden University, Östersund, Sweden; 11 Social Equity and Health Section, Centre for Addiction and Mental Health and Bloomberg Faculty of Nursing and Dalla Lana School of Public Health, University of Toronto, Toronto, Canada; 12 Division of Health Research, Lancaster University, Lancaster, United Kingdom; 13 Indian Institute of Management, Centre for Public Policy, Bangalore, India; 14 Human Early Learning Partnership (HELP), University of British Columbia, Vancouver, Canada

## Abstract

Piroska Östlin and colleagues argue that a paradigm shift is needed to keep the focus on health equity within the social determinants of health research agenda.

**Summary Points**

- Based on extensive review of global evidence, the recommendations of the WHO Commission on Social Determinants of Health highlight the need for strengthening research on health equity with a focus on social determinants of health.
- To do so requires a paradigm shift that explicitly addresses social, political, and economic processes that influence population health; this shift is under way and complements existing research in medicine, the life sciences, and public health.
- Reflecting further synthesis and stakeholder consultations, an agenda for future research on health equity is outlined in four distinct yet interrelated areas: (1) global factors and processes that affect health equity; (2) structures and processes that differentially affect people's chances to be healthy within a given society; (3) health system factors that affect health equity; and (4) policies and interventions to reduce health inequity.
- Influencing regional and national research priorities on equity and health and their implementation requires joint efforts towards creating a critical mass of researchers, expanding collaborations and networks, and refining norms and standards, with WHO having an important role given recent mandates.

## Introduction

A 2009 World Health Assembly resolution on reducing health inequities through action on social determinants of health [1] calls for stakeholders, including researchers and research funders, to give this topic high priority. In 2004, the World Health Organization (WHO) established a Task Force on Research Priorities to outline a global research agenda on equity and social determinants of health. Its 2005 report [2] contributed to the selection of themes for nine Knowledge Networks set up by WHO to support the Commission on Social Determinants of Health (CSDH) during 2005–2008.

CSDH defined health equity as the absence of systematic differences in health, between and within countries, that are avoidable by reasonable action. Using health equity as the foundation of its approach, CSDH concluded [3] that “[s]ocial injustice is killing people on a grand scale” and made three overarching recommendations: improve people's daily living conditions; tackle the inequitable distribution of power, money, and resources; and measure and understand the problem and assess the impact of action. CSDH emphasized that knowledge gaps must not be used as a reason for postponing action on the ample body of evidence already available, but also highlighted the need for ongoing research with a focus on social determinants of health and health equity.

 Subsequently, WHO set up a task force to update the advice provided in 2005, incorporating evidence collected for the CSDH by Knowledge Networks and benefiting from stakeholder consultations on research priorities on equity and health held at seven international meetings during 2007–2009. This article draws from the second task force's longer report [4] completed in 2010, and responds to two questions:

1. In what areas of research could WHO and other development partners concentrate support in order to best advance health equity?
2. What aspects of research, including the development of concepts, methods, norms and standards, and synthesis approaches, could best benefit from global collaboration?

The second task force recommended three key additions: focus on identifying and evaluating policy options, propelled by the search for what works in practice to reduce health inequities; empower research managers, policy makers, and funders to generate national and regional research agendas and fund priorities that address equity and health; and support the strengthening of collaborations, capacities, and methods to do so. Our hope is to help WHO to further advance the health equity agenda, as recently re-articulated in 2010 in World Health Assembly resolution 63.21 on health research [5].

## Advancing Health Equity: A Paradigm Shift in Health Research?

The first wave of contemporary health research focused on medicine and the life sciences, with clinical solutions as a primary endpoint. Although such research remains foundational, understanding the social origins of disease—the “upstream” influences on (ill) health and its distribution [6]—generally and almost unavoidably falls outside the biomedical frame of reference. The past few decades have seen the emergence of a second wave of health research, providing the evidence base for a variety of interventions directed at improving the health of populations rather than individuals, with a large component addressing non-communicable diseases.

The work of CSDH underscores the need for more research on how social, political, and economic processes influence health inequities. We consider this growing field of enquiry [7],[8] as a paradigm shift and a third, complementary, wave of health research. The new paradigm makes explicit that health systems and the people who use them exist within a social context that can powerfully determine peoples' chances to be healthy not only through access to health services, but also through access to a range of other resources, opportunities, and rights: the social determinants of health. Doing research from this perspective involves re-emphasis of older public health traditions and a push for innovative thinking that incorporates a number of distinct strategies and methodologies (Box 1).

**Box 1. Characteristics of Third Wave Health Research Strategies and Methodologies**

- Go beyond the behavioral and other individual determinants of illness.
- Examine the intersections among different social hierarchies, such as class and gender, and their cumulative impacts on health status and health inequities.
- Examine the levels, pathways, and power connections across the “upstream” determinants or root causes of health inequities—that were central to the CSDH's conceptual framework [3]—and the more traditionally investigated determinants of health inequities, such as risk factors or access to care.
- Treat patterns of health inequity as a social reality in their own terms, requiring social (economic, sociological, political, and cultural) explanation that adds on to the aggregation and interpretation of individual biomedical processes and outcomes.
- Consider the dynamic (rather than static) nature of equity in different country contexts, introducing a temporal dimension when investigating social structures, public policies, and impacts over the life course.
- Describe the social institutions and processes that influence the generation and allocation of resources related to health and its social determinants.
- Focus on how the global context affects choices about resource allocation at national and sub-national levels.
- Build on active collaboration among researchers and other knowledge producers from different disciplines.
- Recognize that certain kinds of evidence, such as results from randomized controlled trials, cannot be generated with respect to many interventions that address social determinants of health; therefore, a need exists to embrace diverse methodologies—fit for purpose—including a wide range of study designs, generating qualitative and quantitative data, that provide critical insight on the questions being examined.
- Involve affected populations, which is often essential to appropriate research designs and their execution.

## Research Priorities

Using this frame, we recommend an agenda for research on health equity organized around four distinct yet interrelated areas:

### (1) Global Factors and Processes That Affect Health Equity

“Global health has come to occupy a new and different kind of political space that demands the study of population health in the context of power relations in a world system” [9]. Numerous global processes affect social determinants of health [10]. Global re-organization of production has involved the emergence of an increasingly feminized and informalized global labour market with adverse effects on women's health and their social protection and increases in child labor. Trade liberalization has led to losses of livelihood, sometimes large revenue shortfalls for low- and middle-income countries, increasing privatization of public services such as water, and reduced access to essential medicines. The hyper-mobility of capital has also constrained social policy, as jurisdictions compete for investment, and exposed national economies to the destabilizing effects of disinvestment and financial crises.

It is necessary to improve the evidence base about globalization, not only negative effects, but also positive impacts: for example, expanded social and economic opportunities for women despite harsh working conditions [11]. Comparative cross-national research should be complemented by detailed national case studies that connect household-level impacts with national policies and global forces. Similarly, research on how to redesign institutions for global decision-making—often referred to as “global governance”—is needed so that these institutions address not only trade and economic crises, but other global issues, such as climate change, that have important social and health consequences. The financial crisis of 2008 only underscored this urgency [12].

Globalization is implicated, as well, in many health risks associated with environmental hazards [13]. Potential natural limitations of support for the human species have been widely discussed in recent environmental health fora: our current global trajectories of unsustainable development are important areas for future research.

Rapid urbanization in the developing world is closely connected to globalization: a turning point was reached early in this century, when for the first time a majority of the world's population lived in cities. It is estimated that 1.4 billion people will live in slums in 2020 in the absence of rapid and effective policy interventions [14], creating formidable challenges for reducing health inequities in low- and middle-income countries [15]. Pertinent questions include how global-scale processes lead to social changes that are beyond the reach of local or metropolitan government policies and interventions. Conversely, the emergence of metropolitan areas as global-scale economic actors in their own right potentially offers a new frame of reference for initiatives to reduce health inequities.

Research on globalization and health clearly covers many topics. Building on existing international frameworks and efforts at global health diplomacy, we suggest asking, for example, how the international human rights law framework and recent changes in donor policy, as contained within the Paris Declaration, can shape development assistance and better advance health equity.

### (2) Structures and Processes That Differentially Affect People's Chances to Be Healthy

The social environment in which we live generates unequal distributions of power, wealth, exposures and vulnerabilities to illness. What are the interactions between the axes of social differentiation and how do these contribute to the patterning of inequity at population level [16]? What is the full range of public policies that affect determinants of health like employment relationships and conditions [17] or the operation of gender norms [18]? More specifically, how do economic status, ethnicity, and gender intersect to shape health risks and outcomes? For example, the determinants and consequences of limited to no access to health services often vary by both the gender and class location of sick individuals and their households: research only analyzing class markers can be misleading, as differences across classes can be misinterpreted without gender analysis [19]. How are these intersections affected by the interaction of economic and social policies? Such interactions and their effects frequently begin in early childhood and continue across the life course [20],[21].

Against this background, coordinated and urgent efforts are needed to **shift research from single risk factor analysis to more comprehensive perspectives.** The single risk factor approach fails to uncover multi-causal mechanisms and root causes behind health disparities, and is likely to overlook the accumulation of influences on health over the life course or across generations. The life-course perspective, in turn, requires fundamental rethinking of both research priorities and policy and practice to reflect what is already known about, for example, how material deprivation and stresses associated with subordinate or marginalized social status “cluster cross-sectionally and accumulate longitudinally” [22] and about the underlying biological mechanisms [20],[23]. Nevertheless, it is essential not to lose sight of the importance of acting on what is already known [24],[25]. For example, the links between health and opportunities for productive and fulfilling social activities require integrating occupational health with a broader social analysis.

Systems, institutions, and financing mechanisms for social protection vary widely in their comprehensiveness and in the stages of the life course involved, for example, support for reducing child poverty, unemployment or old-age pensions. Research has been concentrated on high-income countries where the proportion of the working population in the formal labor market is relatively high and coverage of social protection widespread [26],[27],[28]. Even in such countries, much remains to be learnt about how variations in systems of social provision, for example eligibility based on contributions versus universal approaches, operate to influence health. Another important dimension to investigate is the distribution of benefits from public services and their financing sources. In simplest terms, do public expenditures primarily benefit the poor or marginalized, or is their distribution regressive, with the poor disproportionally paying out more than they receive? Understanding the cumulative effects of social protection systems over the life course in a variety of contexts remains important, particularly low- and middle-income countries where systems of social protection are highly diverse and approaches to generate funds remain limited. All countries should monitor and evaluate the gendered health impacts of privatization of social security and pension reform.

### (3) Health Services and Health System Factors That Influence Health Equity

In the past three decades “health sector reform” (HSR) around the world involved increased emphasis on market-based and privately financed solutions. This direction was actively promoted by international financial institutions [29] and exacerbated by domestic austerity programmes during the era of structural adjustment. Available research on HSR suggests that many of the reforms have increased barriers to access to essential preventive services and medical treatments. Crucially, out-of-pocket expenditures for public and private health services continue to drive many families into poverty in low- and middle-income countries [30],[31]. With increased attention to **universal health coverage** [32],[33], a major area for investigation is how to increase access to health services without catastrophic financial burden. Mechanisms that health systems can use to progress towards universal coverage and increase health equity should be evaluated within countries, with evidence synthesized and shared across countries [34]. An important question is why some jurisdictions do far better in providing health services, to a wider range of people in need, than others where public expenditure per capita is comparable. Recognizing the limitations of relying only on supply-side approaches, research needs to generate increased understanding of the value of “demand-side” interventions and approaches to enhance the accountability of health service providers to users [35]. Related, new, or updated methodologies (for example, benefit-incidence analysis, micro-simulation, long-range scenario planning, etc.) could contribute to research on health systems and equity.

Health inequities often cannot be addressed adequately if health systems must be financed only from domestic resources. With much work on identifying resource needs already available, research should **identify sustainable and innovative mechanisms for longer-term and predictable forms of global financing** of health systems in low-income countries. Rapid investigations on how the current financial crisis is affecting public financing for health systems would be timely and practical as inputs to government policy making on health systems and development aid [36]. How are countries or decentralized administrative units coping with increased budgetary pressures and their potential effect on equity? Under what policy and implementation models does decentralization lead to improved local decision-making, net health equity gains, and community empowerment? The recent rise of “medical tourism” also warrants further study of such questions as whether public funds are subsidizing the creation of private, often state-of-the-art hospitals to attract foreign patients and foreign currencies to the detriment of residents' access to health services [37].

Health systems deliver better and more equitably distributed health outcomes when organized around primary health care (PHC) that combines prevention and health promotion with treatment and rehabilitation [32],[34]. Thus, another area for research is how different funding, delivery, and management models of PHC support comprehensiveness of services and equity in access. As PHC principles also include intersectoral approaches, research on how health systems can champion and contribute to actions on social and environmental determinants of health would be particularly useful. Relatedly, of major importance are research and policy that focus on human resources for health. The quality, commitment, and dedication of health workers are critical to the functioning of health systems [38]. The role of women in both formal and informal health services provision is drastically neglected and under-reported, and the gendered nature of human resources for health has not figured largely in health research or policy [39]. Recent assessments indicate that the “brain drain” of providers from low-income countries, especially from those in southern Africa, threatens to precipitate a complete collapse of health systems already stretched to the breaking point by financial constraints and the impacts of HIV and AIDS [38]. Key questions include identifying the most important policy actors and entry points to reduce the health inequities arising from health worker migration patterns.

### (4) From “Problem Space” to “Solution Space”: Effective Policy Interventions to Reduce Health Inequity

Research oriented towards reducing health inequity has until recently focused on what might be called the “problem space.” Building on the foundation of research evidence about causal processes, it is also important to design research that specifically addresses what might be called the “solution space” [40]: the strategic drivers of reductions in health disparities, the differential health effects of public policies, and the comparative effectiveness of options for enhancing equity.

Over the short term, more emphasis is needed on evaluation methodologies that capture contextual and other critical influences, to understand not only how interventions work, but also why they work [41]. Because policies that affect health are often made by finance ministries and not by health ministries, health impact assessments (HIAs) that specifically incorporate equity analysis and apply to policies outside the health system offer a useful basis for integrating the distribution of health outcomes into governmental decision-making [42]. To evaluate impact, a key question is: *How will we know in 20 years which initiatives, by whom, have worked to reduce health inequities within and across countries?* Answering this question requires improved baseline data on health outcomes and social conditions, linked databases, and study designs that enable understanding of complex causality, coupled with research on how policies that do not explicitly target health outcomes affect social determinants of health. Such research, in turn, must rely on a plurality of evaluation methodologies and a broader range of knowledge producers.

#### Knowledge translation to policy makers

 Finally, more attention must be paid to making research accessible and useful to policy makers and other potential users, such as civil society organizations. In the context of what is already known about social determinants of health and working within broader development agendas, making research useful implies norms for data collection and disaggregation [43] and more attention to synthesis of relevant evidence generated outside of disciplines familiar to some mainstream health researchers, for example, in development economics, international political economy, and sociology.

## Next Steps to Advance an Equity-Focused Health Research Agenda

**(a) Building a critical mass of researchers with backgrounds in social sciences and non-medical disciplines**, with experience in a plurality of methods, complementing existing biomedical and biostatistical competencies and in engaging policy makers to further refine research questions. Notably, this will enhance the quality of technical support and policy advice to WHO Member States and enable WHO to function as a more effective advocate.

**(b) Building networks for research support and advocacy and pursuing new research partnerships** focused on social determinants of health and health equity with academic research units, civil society organizations, and other multilateral entities with relevant expertise. Building research partnerships with other UN and development agencies, and with researchers and organizations in low- and middle-income countries, is especially important.

**(c) Establishing and expanding a budget** dedicated to supporting research and research policies related to social determinants of health and health equity. For WHO, this implies mobilizing the resources necessary to support considerable increases in the budget allocation for its strategic objective 7 addressing “the underlying social and economic determinants of health through policies and programs that enhance health equity and integrate pro-poor, gender-responsive, and human rights-based approaches”, as noted in WHO's 2008–2013 medium-term strategic plan [44]. Appropriate resources will further enable intensive efforts across WHO to integrate reduction of health inequities into national and regional research agendas and enable the WHO secretariat to facilitate Member States' requests related to resolution 62.14 [1].

**(d) Ensuring that norms and standards for the monitoring and assessment of health inequalities on multiple dimensions including class, gender, age, and ethnicity are updated** and used in the course of data gathering, statistical analysis, and dissemination to support countries in their efforts and wider global monitoring.

## Conclusion

The report of the CSDH has placed health equity on the agenda of the international community in an unprecedented way, leading to numerous responses. The WHO Region for Europe recently commissioned a European Review of Social Determinants and the Health Divide, to highlight the relevance of the findings of the CSDH and enhance capacities both within and outside the health sector to address health inequities within the region's 53 countries [45]. During the Spanish presidency of the European Union, the government of Spain led the preparation of an expert report [46] on moving forward equity in health and held a European ministerial conference in April 2010, followed by a national commission and report [47] for Spain. Many other countries, notably Brazil, Chile, England [48], and Denmark [49], have begun translating the Commission's findings and recommendations into their national policies, and in some cases, research priorities. These are all positive signs, but with the passage of time comes a serious risk that momentum will be lost. The time to work together and further advance the new paradigm in health research is now.

## Abbreviations

CSDH: Commission on Social Determinants of Health
WHO: World Health Organization
